# Robotisch assistierte Schraubenplatzierung einer Kriechschraube und einer SI-S1-Schraube

**DOI:** 10.1007/s00113-025-01580-z

**Published:** 2025-05-06

**Authors:** Dominik M. Haida, Iris Wagenblast, Stefan Huber-Wagner

**Affiliations:** 1 Klinikum rechts der Isar, Klinik für Unfallchirurgie, Technische Universität München, München, Deutschland; 2 Klinik für Unfallchirurgie, Wirbelsäulenchirurgie und Alterstraumatologie, DIAK Klinikum Schwäbisch Hall, Diakoniestraße 10, 74523 Schwäbisch Hall, Deutschland

**Keywords:** Robotik, Navigation, Becken, Roboterarm, Beckenring, Robotics, Navigation, Pelvis, Robotic arm, Pelvic ring

## Abstract

**Operationsziel:**

Ziel dieser Operation ist es, die vorderen Beckenringfrakturen beidseits sowie die Sakrumfraktur rechts durch eine robotisch assistierte Schraubenosteosynthese zu stabilisieren und damit die Mobilität der Patientin wiederherzustellen.

**Indikation:**

Die Indikation stellt sich durch die sehr starken Schmerzen, der daraus resultierenden Immobilität der Patientin unter konservativen Therapiemaßnahmen und dem Vorliegen einer „fragility fracture of the pelvis“ FFP-II-Fraktur.

**Kontraindikationen:**

Typische Kontraindikationen, wie sie bei dieser Art von Operationen auch bei konventionellen Techniken gibt (v. a. Infektion und Schwellung).

**Operationstechnik:**

Durchgeführt in dem 3D-Navigations-Hybrid-OP „Robotic Suite“ mit den folgenden Komponenten: Navigationseinheit „Curve Navigation System“, fahrbares robotisches 3D Cone Beam CT (CBCT) „Loop-X“, robotischer Arm „Cirq Arm System“ und Wandmonitor „BUZZ“ (Brainlab München, Deutschland).

Die Erläuterung der einzelnen Operationsschritte erfolgt im Video online (Englisch).

**Weiterbehandlung:**

Vollbelastung, Schmerzmedikation nach WHO-Stufenschema und Physiotherapie ab dem ersten postoperativen Tag.

**Evidenz:**

Navigierte und robotische Techniken werden v. a. an der Wirbelsäule verwendet. Am Becken finden diese Techniken ebenso zunehmend Verwendung, wobei auch hier sehr hohe Genauigkeiten erreicht werden können.

**Video online:**

Das Video zur vorgestellten Operationstechnik finden Sie unter folgendem Link (10.1007/s00113-025-01580-z) oder direkt über den unten stehenden QR-Code.

## Abstract (en)

**Objective of surgery:**

The aim of this operation is to stabilize the anterior pelvic ring fractures on both sides and the sacral fracture on the right using robotically assisted screw osteosynthesis and to restore the mobility of the patient.

**Indications:**

The indications arise from the patient’s very severe pain, resulting immobility under conservative treatment measures and a fragility fracture of the pelvis grade II (FFP II).

**Contraindications:**

Typical contraindications for this type of operation are the same as for conventional techniques (mainly infection and swelling).

**Surgical technique:**

Performed in the 3D navigation hybrid operating theatre Robotic Suite with the following components: navigation unit „Curve Navigation System“, movable robotic 3D cone beam computed tomography (CBCT) „Loop‑X“, robotic arm „Cirq Arm System“ and wall monitor „BUZZ“ (Brainlab, Munich, Germany). The individual surgical steps are explained in the video online (English).

**Follow-up treatment:**

Full weight bearing, pain medication according to the World Health Organization (WHO) stage scheme, physiotherapy after the first postoperative day.

**Evidence:**

Navigated and robotically assisted techniques are mainly used on the spine. These techniques are also increasingly being used on the pelvis, whereby very high levels of accuracy can also be achieved.

## Hintergrund

Bei vielen Eingriffen im Bereich der Unfallchirurgie, v. a. im Bereich der Wirbelsäule, zeigen sich die Navigation und Robotik als große Hilfen beim Erreichen von hohen Genauigkeiten [4, 6, 7, 13]. Doch durch den technischen Fortschritt der letzten Jahre erschließen sich neue Eingriffsbereiche wie am Becken und an den Extremitäten, wo ebenfalls erfolgreiche Operationen mit der Unterstützung dieser Techniken durchgeführt werden können [3, 4, 14, 15].

Mit diesem Beitrag wollen wir die Möglichkeiten einer navigierten und robotisch assistierten Operationstechnik im Bereich des Beckens näher erläutern.

## Definitionen und Klassifikationen

Im klinischen Alltag zeigen sich die Auswirkungen der alternden Gesellschaft deutlich. Vor allem in der Unfallchirurgie stehen Insuffizienzfrakturen des Beckenrings stellvertretend für diese Veränderung. Diese Insuffizienzfrakturen werden nur durch ein leichtes Trauma verursacht und stehen oftmals unter dem Einfluss verschiedenster körperlicher Veränderungen oder Veränderungen des Stoffwechsels im Alter [9]. Somit bedarf es oftmals keiner speziellen Umstände, um diese Art von Frakturen zu erleiden. Typischerweise sind Stürze im häuslichen Umfeld Verursacher von Insuffizienzfrakturen des Beckenrings [16]. Bei älteren Patienten können solch vermeintlich harmlose Stürze, extreme lebensverändernde Folgen nach sich ziehen. Neben einer oftmals nicht mehr selbstbestimmten Lebensweise und einem notwendigen Umzug ins Pflegeheim zeigen sich das Erleiden von nosokomialen Infektionen sowie eine hohe Mortalität [2, 16].

Die Frakturen des Beckens können nach der AO Klassifikation (A–C) eingeteilt werden [8]. Für Insuffizienzfrakturen eignet sich jedoch die noch relativ junge, aber bereits etablierte Klassifikation „fragility fractures of the pelvis“ (FFP) besser, da an die einzelnen Kategorien Therapieentscheidungen geknüpft wurden [11, 12]. Eine Einteilung erfolgt hierbei in die Kategorien I–IV, wobei die Lokalisation wie auch die Instabilität (ansteigend von I–IV) durch jede Kategorie berücksichtigt werden. In den einzelnen Kategorien geben die Buchstaben a–c noch eine genauere Abstufung in Bezug auf die Frakturmorphologie.

## Fallbeschreibung

Eine 83-jährige Patientin wurde nach einem Sturz im häuslichen Umfeld, durch den Rettungsdienst in unserer Notaufnahme vorgestellt. Die Patientin war wach und bewusstseinsklar, klagte aber über Schmerzen im Hüftbereich wie auch im Bereich des Beckens. Daraufhin wurde eine Röntgendiagnostik veranlasst. In den Aufnahmen der Beckenübersicht, des Sakrums und der Lendenwirbelsäule (LWS) in 2 Ebenen zeigten sich bereits Frakturen am Os ischii rechts und Os pubis links (Insuffizienzfrakturen). Zur genaueren Diagnostik wurde nachfolgend wurde eine CT-Untersuchung durchgeführt, in welcher eine vordere Beckenringfraktur beidseits und eine Sakrumfraktur rechts (Beckenringfraktur Typ IIb nach FFP-Klassifikation) diagnostiziert wurden [11].

## Operationsindikation

Der initiale Therapieversuch erfolgte primär konservativ mit einer Schmerztherapie nach dem WHO-Stufenschema sowie mit Physiotherapie. Nachdem die Patientin ihre vorherige vorhandene Mobilität aufgrund sehr starker Schmerzen (visuelle Analogskala (VAS) 7) nicht ansatzweise wiedererlangen konnte, wurde gemeinsam mit der Patientin die Entscheidung zur operativen Therapie getroffen.

Dazu gibt die Literatur eine Empfehlung bei FFP-II-Frakturen, eine chirurgische Therapie aufgrund ungenügender Heilungschancen unter konservativen Therapiemaßnahmen zu erwägen [11, 12].

## Operationstechnik

An unserer Klinik werden Eingriffe am Becken regelmäßig in unserem 3D-Navigations-Hybrid-OP „Robotic Suite“ durchgeführt.

Dieser Hybrid-OP besteht aus der Navigationseinheit „Curve Navigation System“, dem robotischen und fahrbaren 3D Cone Beam CT (CBCT) „Loop-X“, dem Wandmonitor „BUZZ“, dem robotischen Arm „Cirq Arm System“ sowie Mixed-Reality-Brillen zur Planung (Brainlab, München, Deutschland).

Als Osteosynthesematerial wurden Schrauben der Fa. Königsee (Allendorf, Deutschland) verwendet. Die verwendete iliosakrale Schraube auf der Höhe S1 (SI-S1-Schraube) besitzt die Maße (Durchmesser × Länge) 7,5 × 85 mm und die Kriechschraube die Maße 7,5 × 85 mm.

Die Patientin ist in Rückenlage auf einem Carbontisch mit den Füßen in Richtung Cone Beam CT (CBCT) gelagert (Video online).

Vor der eigentlichen Operation werden die Schrauben in einem Planungsprogramm auf Basis einer präoperativ angefertigten CT-Bildgebung geplant (0:16).

Zu Beginn der Operation wird die Patientenreferenzeinheit mittels zweier Stäbe an den Beckenschaufeln platziert und ausgerichtet (0:25). Hierbei ist es wichtig, diese vorrausschauend zu platzieren, um eine gute Sichtstrecke zur Kamera zu haben.

Nach der Platzierung der Referenzeinheit können die Vorbereitungen auf den Scan erfolgen (0:53). Erst wird das CBCT positioniert, danach wird die Abbildbarkeit des Eingriffsbereiches mittels einer Feldaufnahme kontrolliert. Ist diese zufriedenstellend, folgt daraufhin die Kollisionsüberprüfung. Ist auch diese abgeschlossen, müssen noch die CBCT-Position im Raum wie auch die Tischposition für nachfolgende Scans gespeichert werden. Für die eigentliche Bildgebung verlässt das Operationsteam den Saal (1. Scan).

Nun folgt die Fusion der präoperativ angefertigten Bildgebung mit Schraubenplanung und der intraoperativ angefertigten CBCT-Bildgebung (1:20). Diese Fusion sollte mit höchster Sorgfalt durchgeführt werden und visuell sowie haptisch mittels Pointer kontrolliert werden. Bei Zweifeln am Fusionsergebnis sollte die Fusion wiederholt werden. Nach der Fusion erfolgt die Registrierung der zu verwendenden Instrumente im System (1:58).

Es folgen nun die robotisch assistierte Planung der Hautschnitte wie auch die eigentliche robotisch assistierte Bohrung der SI-S1-Schraube rechts (02:08). Der Roboterarm wird durch die zwei zu drückenden schwarzen Knöpfe am Kopf in allen Gelenken geöffnet. Dadurch lässt sich der Roboterarm durch Blicken auf den Bildschirm in die Nähe des geplanten Trajektes führen. Erscheint dieses geplante Trajekt in grüner Farbe auf dem Bildschirm, kann der Roboterarm losgelassen werden, daraufhin stellt sich dieser auf die finale und genaue Position des Trajekts ein. Ist dies geschehen, leuchtet der Roboterarm grün. Durch das Einbringen der Bohrhülse in den Arm kann nachfolgend die Markierung des Hautschnitts erfolgen. Nach dem Setzen der Markierung und der Durchführung des Hautschnitts erfolgt eine erneute Anfahrt zur Bohrung nach demselben Prozedere, wie es bereits beschrieben wurde (2:57). Es können nun durch die Bohrhülse die eigentliche Bohrung und das Einbringen des Kirschner Drahtes (K‑Drahts) stattfinden (3:19).

**Abb. 1:**
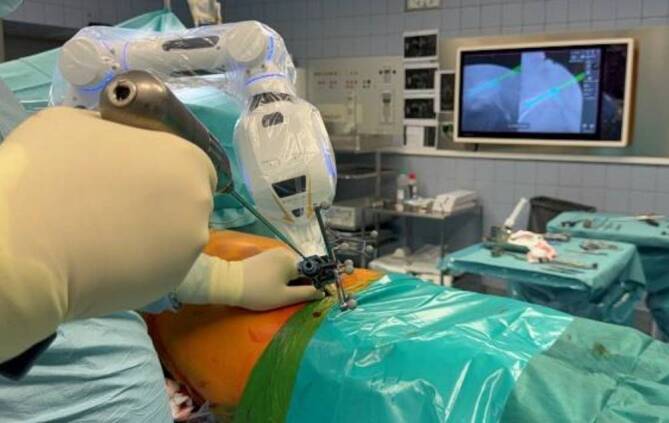
Robotisch assistierte Bohrung der Kriechschraube

Mithilfe des Pointers wird die Hautschnittplanung der Kriechschraube durchgeführt (4:05). Nach dem erfolgten Hautschnitt erfolgen die Bedienung des Roboterarms und die robotisch assistierte Bohrung mit Einbringen des K‑Drahts nach dem gleichen Ablauf, wie bei der SI-S1-Schraube (4:31; Abb. 1).

Nach dem Einbringen beider K‑Drähte für die geplanten Schrauben erfolgt die zweite Bildgebung (2. Scan; 5:42). Davor werden das CBCT und der Tisch auf die gespeicherten Positionen des ersten Scans gefahren. Die Lagekontrolle der eingebrachten K‑Drähte erfolgt unter visueller Kontrolle auf dem Bildschirm (5:46). Es kann zudem ausgemessen werden, ob die geplanten Schrauben ausreichend tief genug einliegen würden.

**Abb. 2:**
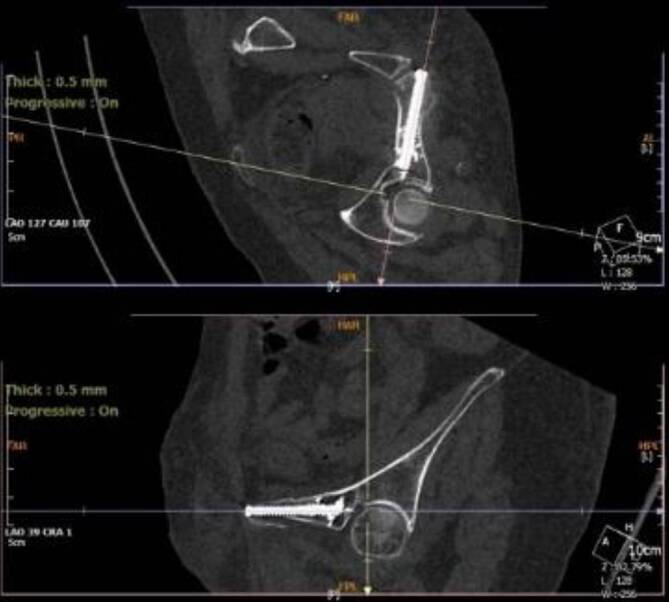
Postoperative Schraubenlage der Kriechschraube

Anschließend erfolgt das Überbohren des K‑Drahtes für die SI-S1-Schraube (6:21). Nun können sowohl die SI-S1-Schraube wie auch die Kriechschraube mittels Schraubendreher und K‑Draht-Führung eingedreht werden (6:30). Anschließend werden beide Schrauben unter C‑Bogen-Kontrolle zementiert (6:49).

Anschließend kann die Naht erfolgen und die Operation beendet werden (nicht im Video gezeigt).

Die Schraubenlage zeigt sich in der postoperativ angefertigten CT Bildgebung sehr zufriedenstellend (06:54; Abb. 2, 3).

**Abb. 3:**
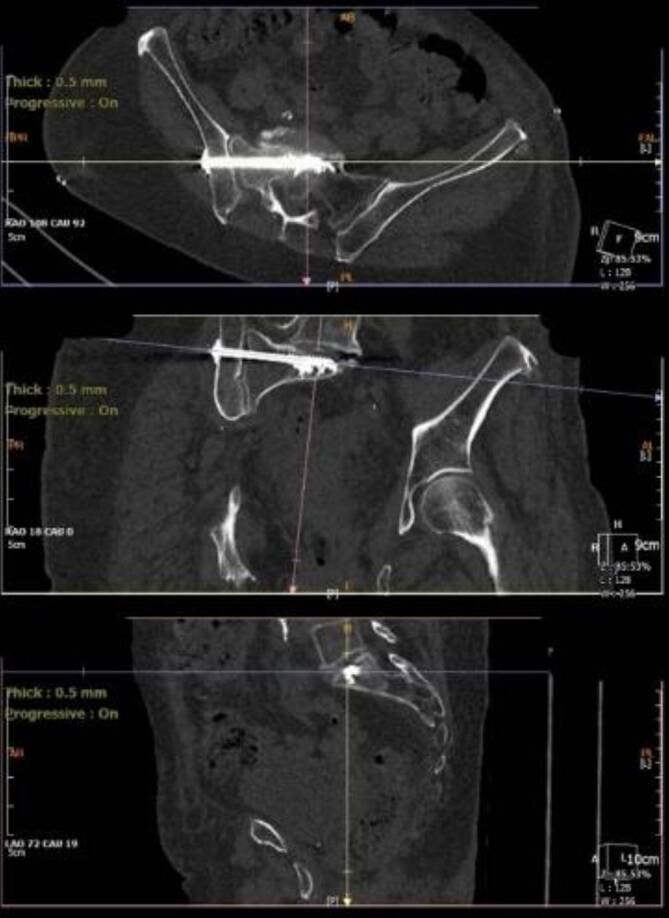
Postoperative Schraubenlage der SI-S1-Schraube

## Fehler, Gefahren und Komplikationen

Die allgemeinen Risiken dieser Art von Operation wie beispielsweise Infektionen und Wundheilungsstörungen entsprechen denen der konventionellen Techniken.

Daneben ergeben sich weiter mögliche Fehlerquellen, welche aber durch gute Vorbereitungen minimiert werden können. Hier zu nennen sind der Ausfall der Navigation und Umstellung auf die konventionelle Technik, die mögliche ungenügende Kontrolle und daraus ungenaue Fusion der präoperativen Planung mit dem intraoperativen CT und Fehler durch menschliche Einflüsse.

Verhindern lassen sich die oben genannten Punkte durch das Beherrschen der konventionellen Technik und dadurch das Schaffen einer Rückfallebene, durch eine Fusion ohne Zeitdruck und adäquate visuelle und haptische Kontrolle mittels Pointer sowie die ausreichende Schulung des gesamten OP-Teams, welches am Eingriff beteiligt ist.

## Evidenz der Technik

Zu der Verwendung von Navigation und Robotik gibt es bereits sehr viele Arbeiten, welche sehr hohe Genauigkeiten unter dem Einsatz dieser Techniken zeigen [1, 4, 5, 10]. Die Eingriffsbereiche werden zunehmend ausgeweitet, sodass neben der Wirbelsäule auch an den Extremitäten und wie in diesem Beitrag dargelegt am Becken, die Verwendung dieser neuen Techniken weiter voranschreitet [3, 4, 14, 15].

Das hier verwendete Videomaterial entstammt einer Routineoperation unserer Klinik. In unserer bereits publizierten Fallserie konnten wir im Bereich der Extremitäten und des Beckens (Acetabulum und Beckenring) eine Genauigkeit von 98,8 % bei der Schraubenplatzierung erreichen [4].

## Fazit für die Praxis

- Eine gute und genaue Planung der Schraubenlagen ist von großer Bedeutung.
- Die intraoperative Kontrolle der CT-Fusion muss mit höchster Sorgfalt durchgeführt werden.
- Das Fusionsergebnis sollte stets visuell wie auch haptisch kontrolliert werden.
- Die intraoperative Kontrolle der Drahtlage bringt zusätzliche Sicherheit.
- Bestimmte, wenig dislozierte Frakturen am Becken können mittels robotisch assistierten und navigierten Osteosyntheseverfahren erfolgreich behandelt werden.

## Supplementary Information

Operationstechnik der robotisch assistierten Schraubenplatzierung einer Kriechschraube und einer SI-S1 Schraube
